# The role and regulatory mechanism of lysosome associated protein transmembrane 4β in tumors

**DOI:** 10.3389/fonc.2025.1552007

**Published:** 2025-03-31

**Authors:** Yuteng Yang, Yumei Li, Yaqi Wang, Xi Chen, Yisong Yao, Dongxian Li, Guohua Yu, Xicheng Song

**Affiliations:** ^1^ The 2nd Medical College of Binzhou Medical University, Yantai, China; ^2^ Department of Otorhinolaryngology, Head and Neck Surgery, Yantai Yuhuangding Hospital, Qingdao University, Yantai, China; ^3^ Shandong Provincial Key Laboratory of Neuroimmune Interaction and Regulation, Yantai, China; ^4^ Shandong Provincial Clinical Research Center for Otorhinolaryngologic Diseases, Yantai, China; ^5^ Yantai Key Laboratory of Otorhinolaryngologic Diseases, Yantai, China

**Keywords:** LAPTM4B, tumor, autophagy, exosome, ceramide

## Abstract

The oncogene *LAPTM4B* (encoding lysosome-associated protein transmembrane-4β), first cloned in hepatocellular carcinoma cells, is located on chromosome 8q22.1 and encodes two isoforms, LAPTM4B-35 and LAPTM4B-24. LAPTM4B proteins have four transmembrane structural domains and are mainly distributed in lysosomal and endosomal membranes of cells. Studies have shown that LAPTM4B is overexpressed in a variety of cancers, in which the genetic polymorphism of *LAPTM4B* is associated with tumor susceptibility. LAPTM4B also regulates various cell signaling pathways, interacts with autophagy-related proteins and ceramides, and regulates the autophagy process and the release of exosomes, which in turn affect the survival and drug resistance of tumor cells. In conclusion, this paper summarizes recent research on LAPTM4B, aiming to explore the role and potential mechanisms of LAPTM4B in a variety of tumors.

## 1 Introduction

Oncogenesis is a complex process characterized by the accumulation of genetic mutations, which leads to dysregulation of cell proliferation, invasion, and metastasis, as well as tumor recurrence, drug resistance, and ultimately poor patient prognosis (1). Various therapeutic approaches targeting oncogenes have been developed; however, the cure rate of cancer using these methods is not satisfactory. In recent years, the *LAPTM4B* gene (encoding lysosome-associated protein transmembrane-4β) has been studied intensively; however, the role and mechanism of LAPTM4B in a variety of tumors remain unclear.

**Figure 1:**
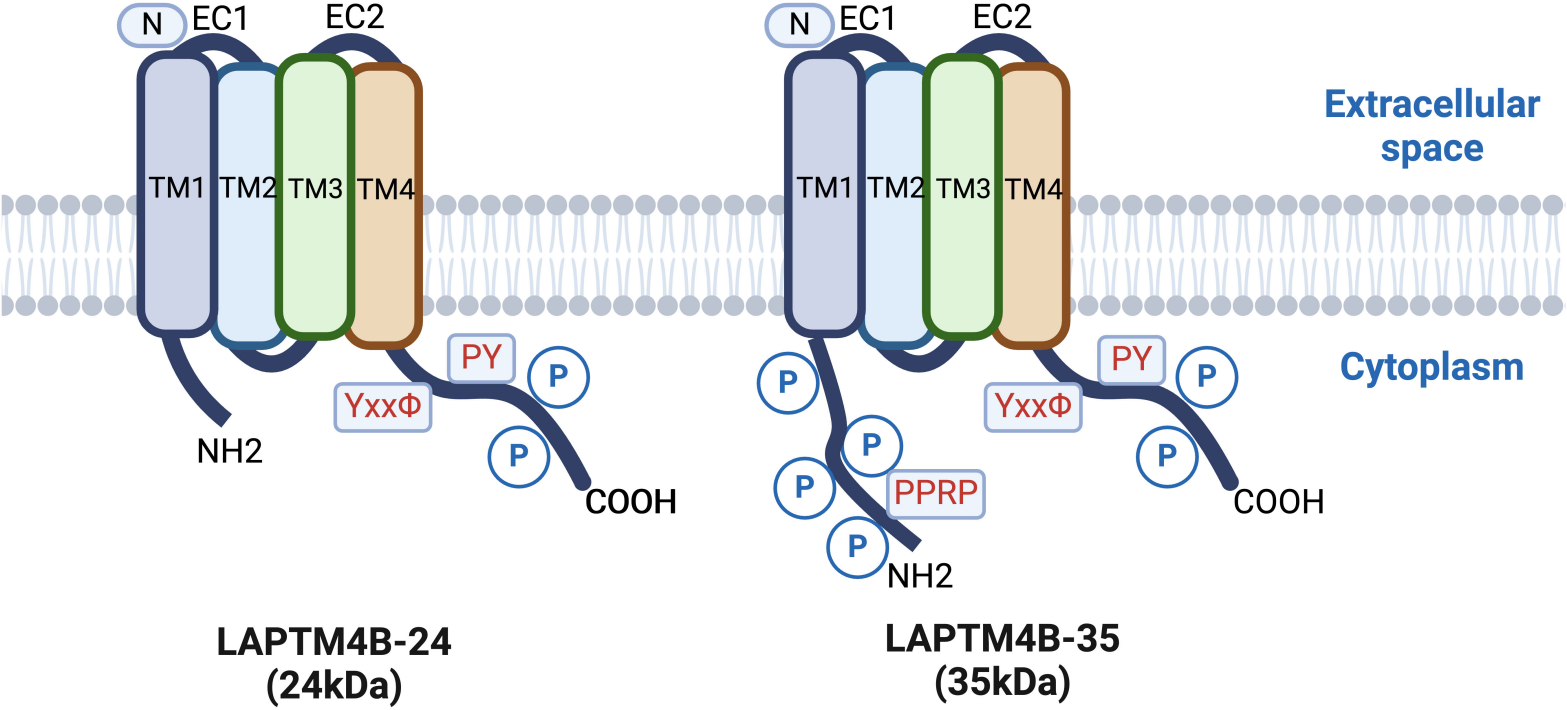
Comparison of LAPTM4B-24 (24kDa) and LAPTM4B-35 (35kDa) protein structures, highlighting key domains and residues relevant to their cellular functions.

**Figure 2:**
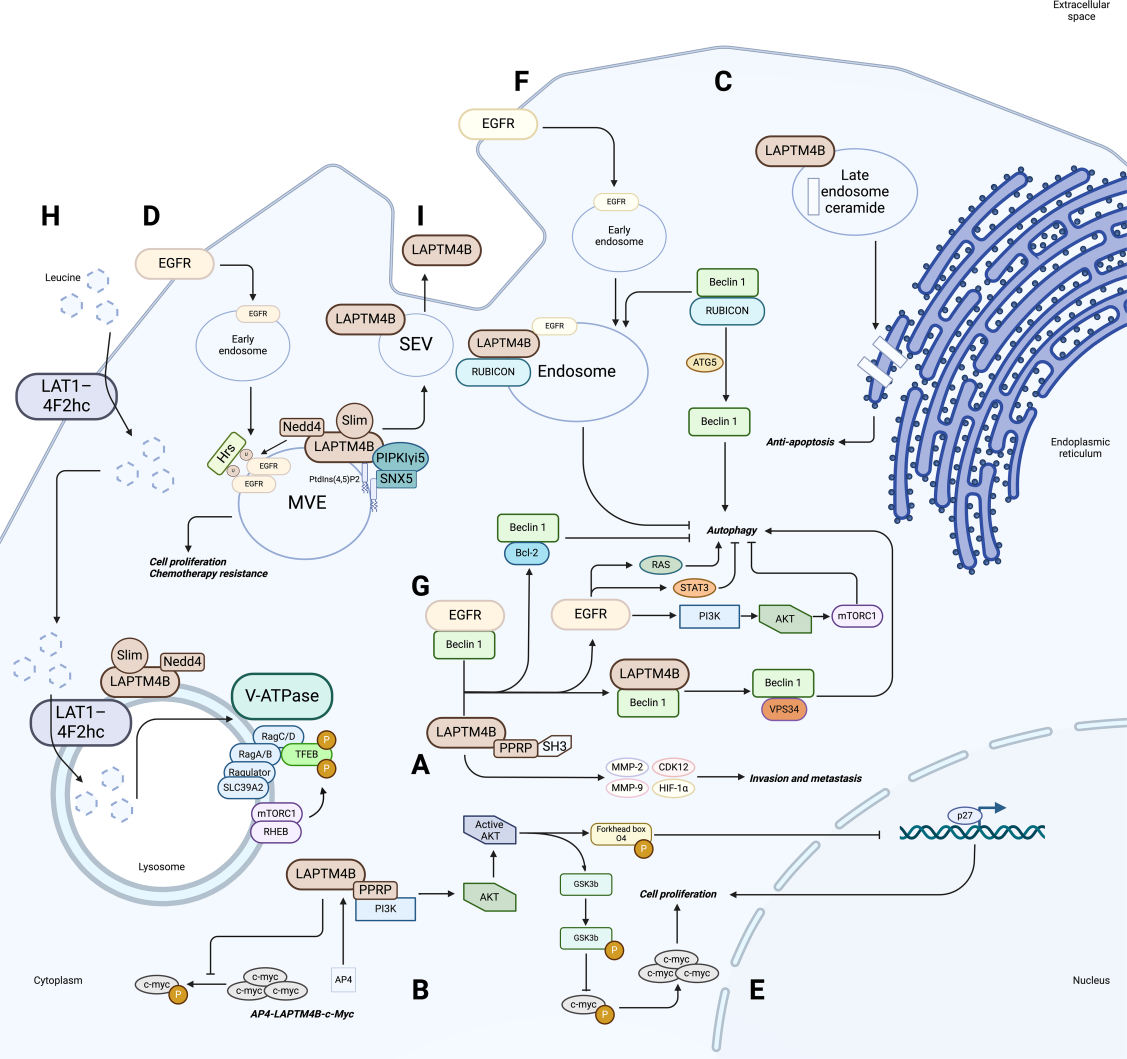
Role and regulatory mechanism of LAPTM4B in tumors. **(A)** Overexpression of LAPTM4B leads to overexpression of MMP-2, MMP-9, CDK12, and HIF-1α, and the PPRP motif of LAPTM4B can interact with these signaling proteins (containing SH3 structural domains) to promote cell invasive metastasis. **(B)** The AP4-LAPTM4B-c-myc axis forms a positive feedback regulatory loop. **(C)** Cells with high levels of LAPTM4B expression exhibit increased clearance of ceramide from late endosomes, thereby increasing cellular sensitivity to ceramide-induced apoptosis. **(D)** LAPTM4B interacts with the E3 ubiquitin ligase Nedd4 and inhibits the binding of Hrs to ubiquitylated EGFR, thereby affecting the endosomal sorting and lysosomal degradation of EGFR, ultimately promoting cell proliferation and chemoresistance. **(E)** LAPTM4B activates the PI3K/AKT signaling pathway through its PPRP motif. Active AKT phosphorylates GSK3b, which attenuates c-Myc phosphorylation, leading to c-Myc accumulation. Active AKT also phosphorylates Forkhead box O4, which in turn affects *P27* gene transcription. **(F)** LAPTM4B interacts with and stabilizes inactive EGFR in endosomes, thereby recruiting ATG5 to dissociate Beclin 1 from the RUBICON - Beclin 1 complex, which triggers autophagy. **(G)** LAPTM4B regulates autophagy through the EGFR signaling pathway. **(H)** The role of LAPTM4B in mTORC1 activation through recruitment of leucine transporters to lysosomes. **(I)** LAPTM4B is a determinant of the sphingolipid profile and membrane properties of small extracellular vesicles (sEVs). LAPTM4B depends on its third transmembrane structural domain, containing the sphingolipid interacting motif (SLim), to be efficiently sorted into the luminal vesicles (ILVs) of multivacuolar endosomes (MVEs), which are released into the extracellular space as sEVs.

LAPTM4B was initially identified in hepatocellular carcinoma (HCC) tissues as potentially involved in hepatocyte proliferation or differentiation, showing high expression in HCC tissue cells, but very low expression in normal adult hepatocytes (2). The *LAPTM4B* gene is located on chromosome 8q22.1 (3, 4) and encodes a protein containing four transmembrane structural domains. Dual in-frame ATG codons located 273 nucleotides apart on the LAPTM4B mRNA initiate translation of two differentially sized protein variants, with electrophoretic mobility corresponding to 35 kDa (LAPTM4B-35) and 24 kDa (LAPTM4B-24) molecular weight markers (5). LAPTM4B-35 differs from LAPTM4B-24 in that it contains an additional 91 amino acid residues at the N-terminus of the proline-rich structural domain (pentapeptide repeat-containing protein (PPRP) (**Figure 1**). The PPRP plays a key role in the proliferative and metastatic potential of tumor cells as a binding site for the SH3 structural domain of certain signaling molecules (**Figure 2A**) (6).

One study found that LAPTM4B mRNA expression was upregulated 13 to 14-fold in the peripheral blood of patients with breast, ovarian, prostate, and colon cancer (7). Previous study evaluated the expression of LAPTM4B mRNA using northern blotting, reverse transcription-PCR, and *in situ* hybridization (8). However, further research remains to be carried out on LAPTM4B and its relationship with carcinogenesis.

## 2 Genetic polymorphisms in *LAPTM4B* are associated with tumor susceptibility

In the normal population, LAPTM4B exhibits three genotypes (^1/1, ^1/2, and ^2/2) and two alleles (LAPTM4B^1 and LAPTM4B^2). The difference is that allele ^1 contains only a single copy of the 19-bp sequence at the 5′-untranslated region (UTR) of the first exon, whereas in allele ^2, this segment is duplicated and tightly tandem repeated (9). These two alleles constitute the *LAPTM4B* polymorphism. Studies have shown that the *LAPTM4B* polymorphism is associated with susceptibility to a variety of solid tumors, such as HCC (9), breast cancer (10–12), non-small cell lung cancer (13), gastric cancer (14), cervical cancer (15), endometrial carcinoma (16), diffuse large b-cell lymphoma (17, 18), gallbladder cancer (19), ovarian cancer (20), papillary thyroid cancer (21), pancreatic cancer (22), and prostate cancer (23). However, not all tumor susceptibility is associated with *LAPTM4B*. For example, susceptibility to esophageal, rectal (24), and nasopharyngeal cancers (25) is not associated with the genetic polymorphism of *LAPTM4B*.

A Chinese population-based mate analysis study found that carriers of allele ^2 had a significantly increased risk of cancer compared with those carrying allele ^1 only (for ^1/2, odds ratio (OR) = 1.55, 95% confidence interval (CI) 1.367–1.758; ^2/2, OR = 2.093,95% CI 1.666–2.629; ^1/2+^2/2, OR = 1.806,95%CI 1.5272.137). Carrying a ^2/2 pure homozygote was significantly associated with cancer risk compared to genotypes carrying the ^1 allele (OR = 1.714, 95% CI 1.408–2.088). Allele ^2 was a risk factor for tumorigenesis (OR = 1.487, 95%CI 1.3391.651) (26). It is noteworthy that activator protein-4 (AP4) is the only factor predicted to bind within the 19-bp polymorphic region of the *LAPTM4B^1* and *LAPTM4B^*2 promoters (27). Thus, the *LAPTM4B* gene polymorphisms correlate with a variety of solid tumors; however, the exact mechanism is not clear and thus more in-depth studies are needed.

## 3 Function of LAPTM4B in tumor cell regulation

### 3.1 LAPTM4B promotes proliferation and metastatic invasion of multiple tumor cells

Increased expression of LAPTM4B-35 and LAPTM4B-24 affects the invasion, metastasis and chemotherapy resistance in a variety of tumor cells, e.g., gastric, breast, colon, ovarian, liver, pancreatic, cervical, prostate, lung, endometrial, and gallbladder cancers (28–43). LAPTM4B with Interacting proteins and potential functions (**Table 1**).

**Table 1:** LAPTM4B with Interacting proteins and potential functions.

References(s)	Interacting/Regulatory partners	Cancer type	LAPTM4B Expression	Function/Bio significances
(32–35, 39, 41)	–	STAD, COAD, OV, PAAD, PRAD, UCEC	↑	Invasion and metastasis
(19, 44)	–	GBC	↑	Cell proliferation Invasion and metastasis
(13, 38)	–	NSCLC	↑	Cell proliferation
(6)	PPRP motifs and SH3 structural domains	Cervical cancer	↑	Invasion and metastasis Chemotherapy resistance
(45)	MMP-2, MMP-9, CDK12, HIF-1α	Cervical cancer	↑	Invasion and metastasis Chemotherapy resistance
(46)	LAT1-4F2hc, mTORC1	Cervical cancer	–	Autophagy
(47)	P-gp	Cervical cancer	–	Chemotherapy resistance Anti-apoptosis
(48)	ATG3	HCC	↑	Autophagy Anti-apoptosis
(48, 49)	Sp1, creb-1 and 19-bp gene sequence	HCC	↑	–
(5, 50, 51)	EMT, AKT, MMP	HCC	↑	Invasion and metastasis
(27)	AP4, c-myc	HCC	↑	Cell proliferation Invasion and metastasis
(52)	SULF2	HCC	↓	Cell proliferation Autophagy
(53)	EGFR, Beclin 1, Vps34	NPC	–	Autophagy
(54)	Caspase-3	SCC	–	Anti-apoptosis
(55)	TM3, Ceramide	SCC	–	Autophagy
(56)	Slim	SCC	–	Exosome release
(54)	Caspase-3	BRCA	↑	Chemotherapy resistance Anti-apoptosis
(47)	PI3K/AKT	BRCA	-	Chemotherapy resistance Anti-apoptosis
(57)	Nedd4	BRCA	↑	Chemotherapy resistance
(58)	PIPKIγi5, SNX5	BRCA	↑	Cell proliferation Chemotherapy resistance
(59)	Ceramide	NB	–	Exosome release
(52)	HOXB4	–	↑	Cell proliferation
(53, 60–72)	HCAL	HCC, OS, LUNG, BRCA, PRAD	↓	Cell proliferation Invasion and metastasis

STAD, Stomach Cancer; COAD, Colon adenocarcinoma; OV, Ovarian Cancer; PAAD, Pancreatic adenocarcinoma; PRAD, Prostate adenocarcinoma; UCEC, Uterine Corpus Endometrial Carcinoma; GBC, gallbladder cancer; NSCLC, non-small cell lung cancer; LUNG, Lung Cancer; HCC, Hepatocellular carcinoma; NPC, Nasopharyngeal carcinoma; SCC, Squamous cell carcinoma; BRCA, Breast Cancer; OS, Osteosarcoma; NB, neuroblastoma .

↑, ↓ These two symbols indicate whether LAPTM4B expression is upregulated or decreased in this tumor.

In nude mouse experiments, after xenografting HCC cells stably overexpressing *LAPTM4B* into the nude mice, the growth of HCC tumor cells was significantly faster than that of the control group, whereas nude mice xenografted with HCC cells stably knocked down for *LAPTM4B* showed lower growth rates than those of the control group. In addition, a 3-(4,5-dimethylthiazol-2-yl)-2,5-diphenyltetrazolium bromide (MTT) assay revealed that the slow growth rate of HCC cells with stable knockdown of *LAPTM4B* was reversed after transfection with a plasmid expressing the LAPTM4B protein (73).

Knocking down *LAPTM4B* in Hela cells and non-small cell lung cancer attenuated cell growth, according to cell counting kit 8 (CCK8) assays (45). In gallbladder cancer, an MTT assay and flow cytometry experiments also confirmed that overexpression of *LAPTM4B* promoted the proliferation of gallbladder cancer cells. In addition, LAPTM4B promotes cancer cell migration by regulating epithelial mesenchymal transition (EMT) (50), protein kinase B (AKT) phosphorylation (5), and matrix metalloprotein (MMP) release (51). Meanwhile, the role of LAPTM4B in promoting cell migration and invasion might be mediated by the PPRP motif of LAPTM4B, a sequence that can interact with proteins containing an SH3 structural domain; thus, participating in multiple signaling pathways to regulate cell migration and invasion (6).

In the previously mentioned positive feedback pathway found in HCC, overexpression of *LAPTM4B* inhibited the phosphorylation of MYC proto-oncogene, BHLH transcription factor (c-myc), leading to its accumulation, and subsequent upregulation of AP4. In turn, AP4 upregulated the transcription of the *LAPTM4B* gene by binding to its promoter, thereby promoting HCC cell proliferation, migration, and invasion (**Figure 2B**) (27, 73, 74). Meanwhile, *LAPTM4B* knockdown inhibited tumor cell migration and invasion, possibly by downregulation of related proteins such as matrix metalloprotein 2 (MMP-2), matrix metalloprotein 9 (MMP-9), cell cycle-dependent protein kinase 12 (CDK12) and Hypoxia-inducible factor 1-alpha (HIF-1α) (**Figure 2A**) (45).

### 3.2 LAPTM4B inhibits apoptosis

In gallbladder cancer cells, overexpression of *LAPTM4B* showed a tendency to inhibit apoptosis, especially under Epirubicin treatment (44). In contrast, doxorubicin-induced apoptosis was significantly increased in *LAPTM4B* knockdown breast cancer cells, suggesting that LAPTM4B might play a role in inhibiting cell survival in breast cancer (75).

In human squamous cell carcinoma and breast cancer cells, stable overexpression of *LAPTM4B* induced sensitivity to caspase-3 activation in response to treatment with anthracyclines or paclitaxel (54). In contrast, tumor cells depleted for LAPTM4B were protected against anthracycline- or paclitaxel-induced Poly (ADP-ribose) polymerase (PARP) cleavage. These results suggested that LAPTM4B might influence cell sensitivity to chemotherapeutic compounds and cell death mechanisms depending on its expression level and the different ways in which the ceramide region is compartmentalized. Cells with high LAPTM4B expression exhibited increased clearance of ceramide from late endosomes (LEs), thereby increasing cell sensitivity to ceramide-induced apoptosis. At the same time, stabilization of late endosomal membranes rendered the cells insensitive to lysosome-mediated cell death (**Figure 2C**) (54). In addition, LAPTM4B depletion leading to the accumulation of sphingolipids in LEs is a potential mechanism to induce increased lysosomal membrane permeability (LMP), leading to cell death (54).

### 3.3 LAPTM4B is associated with tumor drug resistance

In recent years, there has been remarkable progress in research on LAPTM4B in cancer chemotherapy resistance, revealing its complex and multifaceted mechanism of action. For example, *LAPTM4B* knockdown breast cancer cells showed an effective increase in sensitivity to chemotherapeutic drugs such as doxorubicin and zoerythromycin. In addition, *LAPTM4B* knockdown cell lines treated with doxorubicin showed a significant increase in the localization of doxorubicin in the nucleus, suggesting that LAPTM4B might be associated with the nuclear localization of chemotherapeutic drugs (76).

In Hela cells, *LAPTM4B* overexpression increased the efflux of chemotherapeutic drugs, such as paclitaxel and cisplatin, whereas *LAPTM4B* knockdown increased the efficacy of these drugs. The study revealed that LAPTM4B promotes chemotherapy resistance via an interaction with the ATP-dependent membrane efflux transporter protein P-glycoprotein (P-gp) (47). The study revealed that LAPTM4B is not only involved in chemoresistance by affecting drug efflux, but also promotes chemoresistance of cancer cells by activating the phosphatidylinositol 4,5-bisphosphate 3-kinase (PI3K)/AKT signaling pathway and regulating intracellular signaling networks (77–81). In addition, LAPTM4B promotes chemotherapy resistance via an interaction with the ATP dependent membrane efflux transporter protein P-glycoprotein (P-gp) (47).

A recent finding has revealed that LAPTM4B might promote AKT signaling by specifically blocking epidermal growth factor receptor (EGFR) degradation, which provides a novel explanation for its role in chemoresistance. This mechanism involves the interaction of LAPTM4B with the E3 ubiquitin ligase neural precursor cell expressed developmentally down-regulated protein 4 (Nedd4), which inhibits the binding of hepatocyte growth factor-regulated tyrosine kinase substrate (Hrs or endosomal sorting complexes required for transport (ESCRT)-0 subunit) to ubiquitylated EGFR, thereby affecting the endosomal sorting and lysosomal degradation of EGFR (57). In addition, LAPTM4B interacts with the PIP kinase phosphatidylinositol-4-phosphate 5-kinase type 1 gamma (PIPKIγi5) to antagonize the function of LAPTM4B in EGFR sorting by generating phosphatidylinositols (PtdIns) (4, 5), P2 signaling, and recruiting sorting nexin 5 (SNX5) (**Figure 2D**) (58). In addition, AP4 reduces the chemosensitivity of HCC cells through LAPTM4B. AP4 inhibited paclitaxel- and doxorubicin-induced caspase-3-dependent apoptosis by increasing the expression of LAPTM4B. Moreover, AP4 regulates LAPTM4B, and activates the PI3K/AKT signaling pathway and glycogen synthase kinase 3 beta (GSK3b), leading to c-myc accumulation, which amplified the effect of the PI3K/AKT pathway on the drug resistance of hepatocellular carcinoma cells (27).

This finding highlights the complexity of LAPTM4B-mediated regulation of cell signaling and chemotherapeutic drug sensitivity, providing insights and research directions to develop more effective therapeutic strategies in the future. These findings lay a foundation for a more comprehensive understanding of the critical role of LAPTM4B in cancer chemoresistance.

## 4 Molecular regulatory mechanisms of LAPTM4B

### 4.1 Transcriptional regulation of LAPTM4B in tumor cells

The *LAPTM4B* gene is located on chromosome 8q.22.1, a region that contains the *MYC* oncogene (82). LAPTM4B was found to be a direct target gene of AP4 (49), a member of the basic helix-loop-helix leucine zipper (bHLH-LZ) family of transcription factors, which exclusively forms a homodimer that binds to the E-box motif CAGCTG (83). AP4 directly binds to the 19-bp sequence of the promoter of the *LAPTM4B* gene to induce transcription (27). AP4 promotes HCC cell proliferation, migration, and invasion through activation of the PI3K/AKT signaling and caspase-dependent pathways and reduces chemosensitivity through LAPTM4B. Activation of AKT leads to phosphorylation of GSK3b, followed by attenuated c-myc phosphorylation and degradation. In addition, another downstream target of activated AKT, the p27 transcription factor Forkhead box O4 (FOXO4), is phosphorylated, thereby losing its function as a transcription factor (**Figure 2E**) (73).

In addition, sp1 transcription factor and cAMP-responsive element-binding protein-1 (creb-1) are associated with the high expression of LAPTM4B in HCC. The transcription factor sp1 acts upstream of the 19-bp sequence of *LAPTM4B*, and creb-1 acts downstream of the 19-bp sequence to increase the expression of *LAPTM4B (* 49, 84). A mechanistic analysis has shown that sulfhydryl sulfone (SULF2)-induced repression of the *LAPTM4B* gene in HCC cells resulted in decreased autophagosome formation, decreased fusion of autophagosomes with lysosomes, and increased lysosomal membrane permeability. Interference with autophagic flux through inhibition of the SULF2-LAPTM4B axis resulted in decreased LMP, cell viability, and colony formation (85). Notably, *LAPTM4B* was identified as a potential downstream target gene of the homology frame transcription factor homeobox 4 (HOXB4), and its high expression pattern in hematopoietic stem cells and low expression pattern in mature hematopoietic cells might play an important role in stem cell self-renewal and maintenance (52).

**Figure 3:**
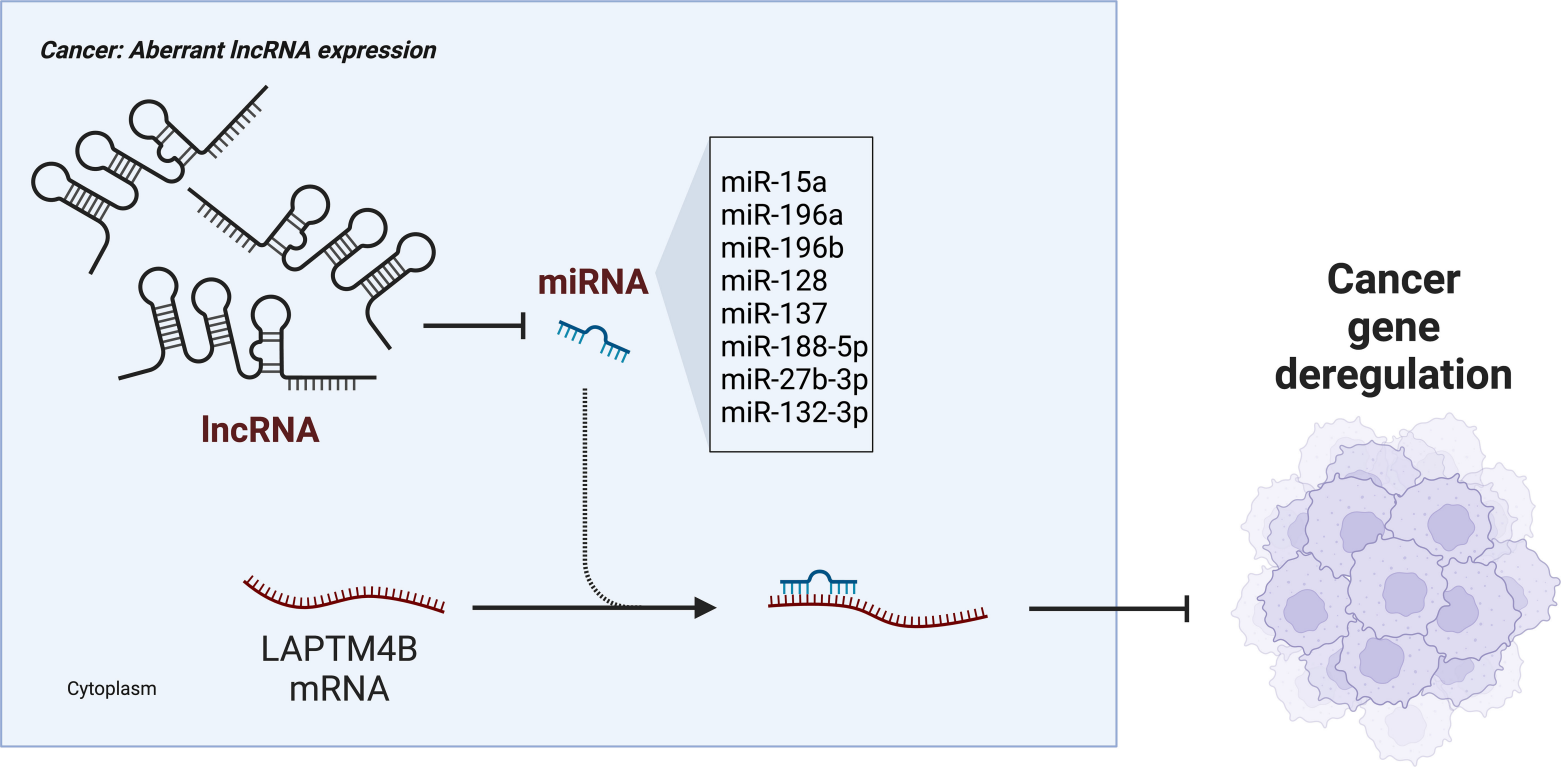
lncRNA-miRNA regulation of LAPTM4B gene expression in cancer.

Many transcripts function as endogenous competing RNAs (ceRNAs) by competitively binding to common microRNAs (miRNAs) (86, 87). In a study of HCC, knockdown of the long noncoding RNA (lncRNA) *HCAL* significantly inhibited the mRNA and protein expression of LAPTM4B in cellular and animal experiments (60). It was also observed that *HCAL* depletion significantly reduced the luciferase activity of a LAPTM4B construct. In addition, *HCAL* downregulation significantly shortened the half-life of *LAPTM4B* mRNA. These results indicated that *LAPTM4B* was a target gene of HCAL. Moreover, by constructing luciferase vectors co-expressing *HCAL* and *LAPTM4B*, it was found that partial ectopic expression of *HCAL* significantly reduced the expression of LAPTM4B. Upregulation of *HCAL* could eliminate this inhibition, suggesting that *HCAL* regulates the expression of LAPTM4B by acting as a ceRNA and by competitively binding to common microRNAs (miR-15a, miR-196a, and miR-196b). Moreover, LAPTM4B overexpression partially rescued the *HCAL* knockdown-induced inhibition of cell migration and invasion, suggesting that *HCAL* regulates cell proliferation, migration, and invasion of HCC cells by modulating *LAPTM4B* expression (**Figure 3**) (60).

LAPTM4B was found to promote RhoA protein stability in osteosarcoma (OS) cells by inhibiting RhoA ubiquitination and RhoA proteasome degradation, which in turn was important in stress fiber regulation (61). Western blotting experiments and dual luciferase assays confirmed that miR-128 and miR-137 directly target *LAPTM4B* mRNA to regulate protein expression. In addition, miR-137 correlated significantly and negatively with LAPTM4B as one of the most down-regulated miRNAs in OS (61, 88, 89). This suggested that the regulation of miR-137 targeting *LAPTM4B* might be involved in OS progression. Similarly, in lung cancer cells, miR-27b-3p can directly target *LAPTM4B* mRNA and inhibit the protein expression of LAPTM4B to inhibit the growth and metastasis of lung cancer cells (62). In breast cancer, miR-132-3p was found to bind directly to the 3’-UTR of the *LAPTM4B* gene and acted at the post-transcriptional level to negatively regulate the expression of LAPTM4B (63). In addition, miR-188-5p could also inhibit the expression of *LAPTM4B* by binding to its 3’-UTR region, thus inhibiting cell proliferation and invasion in prostate cancer (**Figure 2**) (64).

### 4.2 LAPTM4B regulates autophagy

Research over recent years has found autophagy initiation requires the involvement of LAPTM4B. In the environment of metabolic stress, the maturation of autophagic vesicles and autophagic flux can be inhibited by targeting the *LAPTM4B* gene using small interfering RNAs (siRNAs) (48, 65, 76). Nevertheless, the exact mechanism of LAPTM4B’s role in autophagy is incompletely understood.

The upregulation or mutation of LAPTM4B and EGFR in a variety of cancers has attracted much attention because of their association with cancer cell proliferation, survival, drug resistance, and poor prognosis (5, 66). LAPTM4B regulates autophagy through direct activation of ATG3 (encoding autophagy related 3) transcription or through the EGFR pathway, either active or inactive. Research into LAPTM4B in HCC has shown that after silencing of LAPTM4B, the expression levels of many genes changed, including a significant change in the expression of *ATG3 (* 48). In addition, overexpression of *LAPTM4B* led to an increase in the mRNA and protein levels of ATG3. The results also showed that LAPTM4B promoted the growth of HCC under normal conditions, increased cell survival by upregulating the expression of ATG3 under starvation conditions, inhibited apoptosis, and induced autophagy under starvation (48). In addition, under starvation stress conditions, knockdown of *LAPTM4B* resulted in the blockage of autophagosome-lysosome fusion and autophagic lysosome formation. In addition, depletion of LAPTM4B also led to an increase in the number of autophagosomes, but a decrease in autophagic flux, suggesting that LAPTM4B plays a critical role in the later stages of autophagy (48, 76).

LAPTM4B participates in autophagy through the EGFR pathway. It was found that inactive EGFR co-localized with LAPTM4B in early and late endosomes, and in cells under serum-free conditions, the two molecules interacted and stabilized each other (65). As an endosomal protein, LAPTM4B regulates inactive EGFR accumulation in endosomes and inhibits EGF-stimulated luminal sorting and lysosomal degradation of EGFR to promote active EGFR signaling. Specifically, on the one hand, LAPTM4B is required for endosomal accumulation of inactive EGFR and autophagy, and is a cofactor for inactive EGFR-driven autophagy. Inactive EGFR in endosomes interacts with and stabilizes LAPTM4B to recruit ATG5 to dissociate Beclin1 from the RUBICON-Beclin1 complex to trigger autophagy (**Figure 2F**). On the other hand, the expression level of LAPTM4B correlated positively with the level of active EGFR (65). It was also found that LAPTM4B and Beclin1 co-localize in the cytoplasm, where Beclin1 interacts with the N-terminal and C-terminal structural domains of LAPTM4B and competes with EGFR to bind LAPTM4B, which in turn facilitates the initiation of autophagy (53, 67). Therefore, it can be hypothesized that LAPTM4B competes with EGFR to interact with Beclin1 to antagonize autophagy inhibition (68). In nasopharyngeal carcinoma, it was found that LAPTM4B and EGFR form stable endosomes in radioresistant cells and that LAPTM4B interacts with Beclin 1 to promote the initiation of autophagic flux, possibly by promoting the formation of a phosphatidylinositol 3-kinase catalytic subunit type 3 (PIK3C3, also known as Vps34) complex with Beclin 1 (53). However, in gastric cancer, it has been proposed that Beclin 1 interacts with both the N- and C-terminus of LAPTM4B and that this interaction is not dependent on the Vps34 complex (67). In addition, LAPTM4B might regulate autophagy through other EGFR signaling pathways, including PI3K/AKT/mechanistic target of rapamycin kinase (mTOR), EGFR-RAS, EGFR-signal transducer and activator of transcription 3 (STAT3) (**Figure 2G**). PI3K, AKT, and mTOR are downstream molecules in the EGFR signaling pathway, and activated EGFR phosphorylates PI3K and AKT, which activates mTOR and then negatively regulates autophagy (69). EGFR family members activate the RAS/mitogen activated protein kinase (MAPK) pathway, which in turn activates RAF. Activated RAF further promotes autophagy (70–72). In addition, the RAS signaling pathway might promote autophagy by upregulating the expression of ATG5 and ATG7 (90, 91). EGFR inhibits autophagy by interacting with the anti-autophagy proteins B-Cell CLL/lymphoma 2 (Bcl-2) and Beclin 1 (92, 93). In addition, EGFR signaling inhibits autophagy by interacting with Protein Kinase R (PKR) through the SH2 structural domain of cytoplasmic STAT3 (94, 95).

### 4.3 LAPTM4B regulates the mTORC1 signaling pathway

It has been proposed that ceramide sensitizes cancer cells to chemotherapy-induced death (96–98). Nevertheless, cells deficient in acidic ceramidase (ASAH1) and LAPTM4B had higher ceramide levels than cells deficient in ASAH1 alone. However, at this point the cells are desensitized to drug-induced apoptosis and the pro-apoptotic effect through the accumulation of ceramide on the endoplasmic reticulum (ER), which was counteracted by *LAPTM4B* silencing after deletion of ceramide transfer protein (CERT), resulting from the reduction of ceramide reaching the ER after LAPTM4B silencing (54).

Studies of the interaction of LAPTM4B with ceramide have revealed that the third transmembrane region (TM3) of LAPTM4B contains a ceramide-binding site that consists of a sphingolipid-binding motif and a neighboring aspartic acid residue (99, 100)(**Figure 1**). By interacting with this motif, ceramide regulates the conformation of LAPTM4B, making it more likely to bind to the amino acid transporter protein heavy chain 4F2hc, thereby affecting the mechanistic target of rapamycin complex 1 (mTORC1) signaling pathway (54). In contrast, aspartic acid residues in the transmembrane region provide LAPTM4B with functional flexibility, allowing LAPTM4B to reduce TM3 bending in the presence of ceramide, thereby facilitating its binding to 4F2hc. This binding enhances mTORC1 activity to promote cellular nutrient signaling (46, 55).

In studies on the binding of LAPTM4B to the leucine transporter protein (L-type amino acid transporter 1 (LAT1)-4F2hc), LAPTM4B was able to recruit LAT1-4F2hc to lysosomes, as determined by mass spectrometry analysis and immunoprecipitation experiments. The localization of LAT1-4F2hc to the lysosome by LAPTM4B resulted in Leu entry (101, 102). In contrast, knockdown of *LAPTM4B* reduced the lysosomal localization of LAT1-4F2hc, indicating its critical role in Leu entry. Moreover, LAPTM4B promotes Leu uptake into lysosomes by recruiting LAT1-4F2hc to lysosomes and activating the lysosomal membrane protease (V-ATPase) inside lysosomes (inside-out activation) (103), which leads to Ragulator, RagA/B-GTP, and mTORC1 activation via Rheb-GTP, which in turn stimulates mTORC1 activation (**Figure 2H**) (46).

### 4.4 LAPTM4B regulates ceramide-induced exosome release

Exosomes are nanosized extracellular vesicles that originate from endosomes. They release endocytic vesicles (intraluminal vesicles (ILVs)) into the extracellular space through the fusion of multivesicular bodies (MVBs) with the plasma membrane (104). Exosomes can carry proteins, lipids, and RNA, being involved in intercellular molecular communication and waste removal (105).

It was found that in neuroblastoma cells, LAPTM4B binds to ceramide and facilitates the translocation of MVBs to the plasma membrane, which in turn increases the release of exosomes. Exogenous ceramide can enter neuroblastoma cells via the endocytosis pathway and induces exosome secretion. It was found that knockdown of *LAPTM4B* completely inhibited the ceramide-mediated increase in exosome release. In addition, ASAH1 causes ceramide to accumulate intracellularly and increases exosome production in a LAPTM4B-dependent manner (106). LAPTM4B also binds to ceramides of long-chain fatty acids through their sphingolipid-binding structural domain, accelerating the transport of MVBs to the plasma membrane and enhancing exosome secretion (59). Studies found that LAPTM4B contains a functional sphingolipid interaction motif (SLim) in its third transmembrane structural domain (TM3) (55, 99), and that SLim acts to efficiently sort LAPTM4B into the membrane of ILVs for extracellular release. These studies also revealed that LAPTM4B was secreted from human cells in small extracellular vesicles (sEVs) both *in vivo* and *in vitro*. LAPTM4B regulates the glycosphingolipid and ether lipid composition of sEVs in a SLim-controlled manner and modulates EV membrane properties (**Figure 2I**) (56). In addition, exosomes isolated from HCC cell culture supernatants contained LAPTM4B (107).

## 5 Potential of LAPTM4B as a therapeutic target

Although multiple molecularly targeted agents have been developed, some targeted therapies might be ineffective; therefore, additional targets, such as LAPTM4B, are needed to treat cancer. EGFR tyrosine kinase inhibitors (EGFR-TKIs) block the cellular functions mediated by EGFR kinase signaling in non-small cell lung cancer, but they also activate inactive EGFR in autophagy, which may provide a survival advantage and induce TKI resistance in cancer (108). Thus, co-targeting EGFR and other molecules might be a promising strategy to overcome TKI resistance in cancer. LAPTM4B, an oncoprotein that promotes active EGFR signaling in cancer cells and is required for the autophagy process induced by inactive EGFR, might represent a synergistic targeting molecule in cancer therapy (109).

Inhibiting LAPTM4B activates AKT signaling and inhibits cancer cell proliferation (73). Disrupting the interaction between LAPTM4B and SH3 domain-containing proteins controls cancer invasion and metastasis (6). Expression of LAPTM4B reduces the output of advanced endosomal ceramide and promotes apoptosis in cancer cells (54). LAPTM4B promotes drug release through the efflux pump P-gp, which stimulates multi-drug resistance in cancer cells (47). Thus, it is evident that LAPTM4B plays a key role in tumors and offers possibilities for cancer therapy.

A study reported the design and synthesis of the far infrared/near infrared (FR/NIR) fluorescent lamp probe DBT-2EEGIHGHHIISVG, which specifically displays the LAPTM4B protein in cancer cells and tumor-bearing mice. The probe DBT-2EEGIHGHHIISVG enables the targeted visualization of LAPTM4B in human HCC cells, and selective and high-contrast imaging of LAPTM4B protein-expressing tumor tissues in live mice, which has the potential to make LAPTM4B useful in the treatment of HCC (110).

Ethylglyoxal bisthiosemicarbazon (ETS) specifically killed HCC cells by inhibiting the phosphorylation of the Tyr285 of LAPTM4B-35, which is involved in the activation of the PI3K/AKT signaling pathway induced by LAPTM4B-35 overexpression (111). In addition, ETS reversed the effect of LAPTM4B-35 overexpression on the levels of c-myc, B-Cell CLL/lymphoma 2 Bcl-2, BCL2-associated X protein (Bax), cyclinD1, and p-AKT molecules in HCC cells, marking LAPTM4B-35 as a candidate for targeted therapy in HCC (112).

## 6 Conclusion and prospective

The important role of LAPTM4B in cancer biology is increasingly recognized. LAPTM4B is overexpressed in a variety of tumors and affects cell proliferation, migration, invasion, and drug resistance. Its genetic polymorphisms have been associated with susceptibility to several cancers, highlighting its potential as a biomarker in cancer risk assessment. The ability of LAPTM4B to modulate key signaling pathways, such as PI3K/AKT and EGFR, and affect autophagy and chemoresistance, and its ability to interact with autophagy-associated proteins and ceramides, further illustrates the complexity of the role of LAPTM4B in tumor cell survival. In addition, LAPTM4B’s interaction with the P-gp efflux pump enhances chemoresistance, while its modulation of autophagy and apoptotic processes highlights its potential as a therapeutic target. Tumor growth and sensitivity to chemotherapy can be modified by altering LAPTM4B expression, making it a promising target for innovative cancer therapies.

However, the exact molecular mechanisms by which LAPTM4B affects cancer progression remain a focus of future research. Understanding the role of LAPTM4B in different types of cancer is essential to develop targeted therapies. Investigating polymorphisms in the gene will also allow for personalized treatment strategies based on individual genetic profiles. The development of LAPTM4B inhibitors or modulators could provide new avenues for cancer treatment, especially for tumors that have not responded to conventional treatments. Combining LAPTM4B-targeted therapies with existing therapies could improve efficacy and overcome drug resistance. In addition, the potential use of LAPTM4B as a diagnostic marker warrants further exploration. Techniques to visualize LAPTM4B expression *in vivo* could facilitate early cancer detection and monitoring of treatment responses.

In conclusion, the multiple roles of LAPTM4B in tumorigenesis present both challenges and opportunities. Improving our understanding of this protein might lead to major breakthroughs in cancer therapy and holds promise for improved outcomes for patients with drug-resistant cancers.
